# Effects of dopamine D2/D3 receptor antagonism on human planning and spatial working memory

**DOI:** 10.1038/tp.2017.56

**Published:** 2017-04-25

**Authors:** M Naef, U Müller, A Linssen, L Clark, T W Robbins, C Eisenegger

**Affiliations:** 1 Department of Economics, Royal Holloway, University of London, Egham, UK; 2 Behavioural and Clinical Neuroscience Institute, Department of Psychology, University of Cambridge, Cambridge, UK; 3 Adult ADHD Service, Barnet Enfield Haringey Mental Health NHS Trust, London, UK; 4 Department of Neuropsychology and Psychopharmacology, Maastricht University, Maastricht, The Netherlands; 5 Centre for Gambling Research at UBC, Department of Psychology, University of British Columbia, Vancouver, BC, Canada; 6 Neuropsychopharmacology and Biopsychology Unit, Department of Basic Psychological Research and Research Methods, Faculty of Psychology, University of Vienna, Vienna, Austria

## Abstract

Psychopharmacological studies in humans suggest important roles for dopamine (DA) D2 receptors in human executive functions, such as cognitive planning and spatial working memory (SWM). However, studies that investigate an impairment of such functions using the selective DA D2/3 receptor antagonist sulpiride have yielded inconsistent results, perhaps because relatively low doses were used. We believe we report for the first time, the effects of a higher (800 mg p.o.) single dose of sulpiride as well as of genetic variation in the DA receptor D2 gene (DA receptor D2 Taq1A polymorphism), on planning and working memory. With 78 healthy male volunteers, we apply a between-groups, placebo-controlled design. We measure outcomes in the difficult versions of the Cambridge Neuropsychological Test Automated Battery One-Touch Stockings of Cambridge and the self-ordered SWM task. Volunteers in the sulpiride group showed significant impairments in planning accuracy and, for the more difficult problems, in SWM. Sulpiride administration speeded response latencies in the planning task on the most difficult problems. Volunteers with at least one copy of the minor allele (A1+) of the DA receptor D2 Taq1A polymorphism showed better SWM capacity, regardless of whether they received sulpiride or placebo. There were no effects on blood pressure, heart rate or subjective sedation. In sum, a higher single dose of sulpiride impairs SWM and executive planning functions, in a manner independent of the DA receptor D2 Taq1A polymorphism.

## Introduction

The role of the dopaminergic system in modulating cognitive functions within the prefrontal cortex and striatum is well established.^1, 2, 3, 4^ The concept of fronto-striatal circuitry emphasizes the functional inter-relationship between the prefrontal cortex and the striatum, with the latter influencing cortical higher-order cognitive functions and vice versa.^5, 6^ Cognitive functions such as planning and working memory depend critically on dopamine signalling within this circuit. This has been shown by psychopharmacological drug challenges, genetic studies and research on diseases that affect fronto-striatal dopamine (DA) levels.^7, 8, 9, 10, 11, 12, 13^

Although DA D2 receptors occur at lower density in the prefrontal cortex than DA D1 receptors, D2 receptors are nevertheless implicated in planning and working memory. For instance, administration of the DA D2 agonist bromocriptine enhanced performance on a delayed-response working memory task, whereas low doses of the DA D2 antagonist haloperidol impaired performance.^14, 15^ Furthermore, a relationship between striatal DA D2 receptor density and planning accuracy was observed in Huntington's disease patients,^16, 17^ suggesting that the dopaminergic system exerts part of its modulatory role on planning and working memory performance via the DA D2 receptor.

The DA D2/D3 antagonist sulpiride has been investigated using relatively low doses of 200 and 400 mg.^7^ Sulpiride was found to cause a dose-dependent impairment in short-term spatial location memory, as well as impaired planning in the most difficult stages of the one-touch Tower of London task. In a subsequent pharmaco-PET study, 400 mg of sulpiride had no effect on spatial working memory (SWM), and paradoxically improved planning performance.^8^ This effect was paralleled by a decrease in regional cerebral blood flow in the caudate.^8^ In another study^18^ using the self-ordered SWM task from the Cambridge Neuropsychological Test Automated Battery (CANTAB), 400 mg of sulpiride did not affect performance, but puzzlingly, others even reported improved accuracy of working memory following the same dose of sulpiride.^19^

One account for these discrepant results is that perhaps 400 mg sulpiride does not result in sufficient occupancy of postsynaptic DA D2 receptors to reliably impair executive functions. Furthermore, it has been observed that low doses of amisulpride (similar to sulpiride, both being selective for DA D2/3 receptors) exert a greater functional blockade of cortical and limbic, rather than striatal, DA D2 receptors.^20, 21^ DA release may even increase in these regions as a consequence of presynaptic DA D2 autoreceptor blockade.^22^ Overall, the causal role of postsynaptic DA D2 receptors in planning and SWM in healthy humans remains elusive.

To achieve a sufficient blockade of postsynaptic DA D2 receptors within the fronto-striatal circuitry, higher doses of sulpiride may have to be administered. Previous studies have shown that a single dose of 400 mg sulpiride occupies roughly 30% of striatal DA D2 receptors,^23^ whereas an 800 mg dose results in roughly 60% occupancy levels, yet still without causing demonstrable side effects in healthy volunteers.^24, 25^ We therefore used a dose of 800 mg p.o. in the present study.

Finally, although sulpiride does not possess significant binding to α-adrenergic, histaminergic or serotoninergic receptors, it nevertheless does not discriminate between the DA D2 and D3 receptors. As the anatomical distribution of these two receptors is only partially overlapping, a pharmacogenetic study design^24, 26, 27, 28^ may enable more specific inferences to be drawn regarding the role of the DA receptor D2 in planning and working memory performance. A relevant candidate genetic variation in this context is the DA D2 receptor Taq1A polymorphism, as its minor A1 allele has been associated specifically with a reduction in striatal DA receptor D2 density of up to 30 percent.^29, 30, 31, 32, 33^ Based on this evidence, one might expect A1+ volunteers to be disproportionately sensitive to DA receptor D2 antagonism in terms of behavioural impairments in planning and working memory performance.

We hypothesize that a single dose of 800 mg sulpiride administered p.o. to healthy volunteers induces impairments in working memory and planning performance, compared with placebo. We also predict this impairment to be most pronounced in volunteers carrying the minor A1+ allele of the DA receptor D2 Taq1A polymorphism.

## Materials and methods

### Volunteers

Seventy-eight healthy men aged between 19 and 44 years (mean=32.1) participated. All were recruited from Cambridge BioResource, a large community-based panel of volunteers for research linking genotype to phenotype (http://www.cambridgebioresource.org.uk). All volunteers are right-handed European or North American Caucasians. Volunteers were stratified based on their DA receptor D2 Taq1A genotype, with one group consisting of individuals carrying one or two copies of the A1 allele and the other group consisting of A2 allele homozygotes. All volunteers were task-naive and none had participated in previous psychoactive drug studies.

Volunteers' mental and physical health was screened before genotyping using a detailed medical history questionnaire used by Cambridge BioResource. This revealed no history of neurological disease or psychiatric disorders. In addition, the psychiatrist on site performed another structured interview, confirming that volunteers had no significant general psychiatric, medical or neurological disorder and were not currently taking any prescription medicine, nor drugs of abuse. All volunteers were required to perform an alcohol test on arrival at the lab using a commercially available breath alcohol analyser. This confirmed that no volunteer had consumed alcohol on the study day.

The study was performed in accordance with the Declaration of Helsinki and approved by the National Research Ethics Committee of Hertfordshire (11/EE/0111). All volunteers were included in the study only after having provided written informed consent. For three volunteers, data collection was unsuccessful: one felt uncomfortable in the testing room (sulpiride group A1−), and two non-native English-speaking volunteers (placebo group A1−, sulpiride group A1−) did not sufficiently understand the instructions for the CANTAB tasks. In addition to the working memory and planning tasks reported here, volunteers also completed an incentivized reinforcement learning task^24^ and incentivised social interaction tasks (assessing negative and positive reciprocity). The working memory and planning tasks reported in this paper were not incentivized; volunteers received a flat fee of £50 for participation in the study, plus any additional earnings from the incentivized tasks. Verbal intelligence quotient (IQ) estimates were calculated for all volunteers (National Adult Reading Test;^34^ mean=119.8±7.33; range=101–129). A technical fault led to the omission of one further volunteer on the SWM task (sulpiride group A1+).

### Experimental design

We used a between-subject, double-blind, placebo-controlled design, where 78 volunteers were randomized to receive either a single oral dose of 800 mg sulpiride or placebo. The volunteers were stratified based on their DA receptor D2 Taq1A genotype, yielding the following four groups: A1+ volunteers who were administered sulpiride (*n*=21) and A1+ volunteers who were administered placebo (*n*=17); as well as A1− volunteers who were administered sulpiride (*n*=19) and A1− volunteers who were administered placebo (*n*=21). There were no differences across the four groups with regard to age (*P*-values >0.49), body mass index (*P*-values >0.24) or IQ (*P*-values >0.42).

### Procedure

On the study day, volunteers arrived at the lab between 0830 h and 1000 h. At the start, volunteers completed two questionnaires for assessing current mood (visual analogue scale). Then, pulse rate and blood pressure were measured and blood samples (10 ml) were taken. All volunteers then received either a sulpiride or placebo capsule, which was administered orally. After ingesting the pill, volunteers passed a waiting period in individual rooms. While waiting, volunteers were allowed to read newspapers. In line with a previous study,^8^ the planning task was administered 3 h after capsule ingestion to coincide with the time window of maximal sulpiride effects. Before the task started, volunteers had to complete a comprehensive side-effect questionnaire,^35^ current mood, blood pressure and pulse rate were measured and a second blood sample was taken (Supplementary Table 1). The SWM and one-touch stockings of Cambridge (OTSOC) tasks were presented on computers and responses were registered via touch-sensitive screens. At the end of the experiment, volunteers were asked to guess whether they had received the sulpiride or the placebo pill (Supplementary Table 1).

### CANTAB SWM task

The SWM is a self-ordered search task, which requires volunteers to search through a spatial array of 4, 6, 8, 10 or 12 coloured squares (boxes) for a ‘token' that is hidden in one of the boxes. Volunteers touch a box to reveal whether the token is in the box or not. Once a token is found, the search starts again, but is no longer completely random. Volunteers know that no token will be hidden in a box where a token was previously hidden. Thus, each round fewer boxes are possible candidates; each round, volunteers have to remember more boxes that are no longer ‘in the game'. In this test, volunteers have to use mnemonic information to work towards a goal. Between-search errors are ‘forgetting' errors committed when a box that has previously been successful is revisited during a subsequent search. Within-search errors entail revisiting a box within a search, that is, the number of times a volunteer revisits a box already found to be empty during the same search. An efficient strategy for this problem is to start each search sequence with the same box. Our strategy score is quantified as the number of times the volunteer starts a search sequence from a different box (thus a higher strategy score represents inefficient strategy use). The strategy score is typically correlated with working memory errors, but strategy is specifically impaired in patients with frontal (but not temporal) lobe injury.^36^ Volunteers did two practice searches with three boxes each. The practice searches were completed immediately before the main SWM task, and successful solving of these practice searches was a requirement for progressing onto the main test. The main task consisted of fifteen problems in total, three for each of the five difficulty levels.

### CANTAB OTSOC task

We investigated planning using a modified version of the Tower of London task, the OTSOC from the CANTAB (Cambridge Cognition, http://www.camcog.com). In this modified version of the task, volunteers are required to determine the minimum number of moves needed to solve the problem without actually moving any of the balls. This modification forces volunteers to plan the solution in full before initiating a response. This ensures actual planning and enables an improved investigation of the specific relation between the time to initiate the first response (response latency), the problem difficulty and the number of attempts to solve the problem (accuracy).

In the OTSOC, volunteers were first presented with two displays on a computer screen, each showing three coloured balls arranged within three stockings. The challenge was to match the lower to the upper display and to achieve this with the least possible number of moves. The difficulty varied from one to six moves needed to solve a problem. Volunteers were not required or even able to physically move the balls to replicate the upper display. They just had to select the minimum number of moves needed from a list of seven possibilities displayed at the bottom of the screen. They were allowed to take as many attempts as needed to solve the problem. The number of attempts to solve the problem (accuracy) and the time taken to initiate the first response (response latency) were recorded. To confirm that volunteers understood the instructions, they had to successfully complete four practice trials immediately before the main OTSOC task started. The main task consisted of four problems for each of the six difficulty levels, resulting in twenty-four problems in total.

### Prolactin level assessment

Plasma prolactin level elevation is considered to be an indicator of postsynaptic dopamine receptor antagonism.^37, 38^ Postsynaptic dopamine blockade is predicted to elevate prolactin levels at the second time point, 3 h after capsule ingestion.^39, 40^ The prolactin level was measured using a commercial immunoradiometric assay (MP Biomedicals, Santa Ana, CA, USA). The intra- and inter-assay coefficients of variation were 4.2% and 8.2%, respectively, and the limit of detection was 0.5 ng ml^−1^.^24^

### Visual analogue scales and side-effects questionnaire

The visual analogue scale^41^ was used to assess volunteers' current mood state at baseline and 3 h after sulpiride/placebo administration. The original visual analogue scale contains 16 scales. In the present study, we investigated alertness, calmness and contentedness.

Side effects were recorded using a drug effects questionnaire (neurovegetative list)^35^ 3 h after sulpiride/placebo administration.

### Statistical analysis

Statistical analysis was performed using the software package STATA. Differences across groups concerning age, body mass index, general IQ and verbal IQ were analysed using *t*-tests. Concerning the control variables current mood, side effects, blood pressure, pulse rate and prolactin level, we used nonparametric tests such as the Mann–Whitney and the Wilcoxon signed-rank test.

To calculate the standard errors used in the figures, we ran ordinary least squares regressions with the variable on the vertical axis as dependent variable and the variable on the horizontal axis as explanatory variable. We ran such a regression for each subgroup we report in the figures. To take into account the repeated measurement, standard errors were clustered on individual level. The clustered standard errors are also robust to some minor misspecifications such as minor problems about normality, heteroscedasticity or some observations that exhibit large residuals, leverage or influence.

To analyse the effects of sulpiride and genotype on OTSOC and SWM variables of interest (including the practice trials), we conducted a repeated-measures analysis of variance (ANOVA), with task difficulty level as the within-subject factor and sulpiride treatment and genotype as between-subject factors, as well as all interactions between these variables. In Supplementary Tables 2 and 3, we report the full results for the four ANOVAs conducted, whereas in the main text we do not always report all the variables in detail.

To assess the relationship between accuracy and response latency in OTSOC, we used ordinary least square regressions with accuracy as dependent variable and response latency, sulpiride and their interaction as explanatory variables. In Supplementary Table 4, we report the full results for the three regressions conducted. We have excluded two outliers in the regression regarding the relationship between accuracy and response latency, and they are labelled in Figure 4.

To normalize response latency distribution, the data were log-transformed and divided by 1000.^42^ Significant differences are reported as *P*<0.05. Results do not change qualitatively if IQ is included as a control variable.

## Results

### SWM task

**Figure 1:**
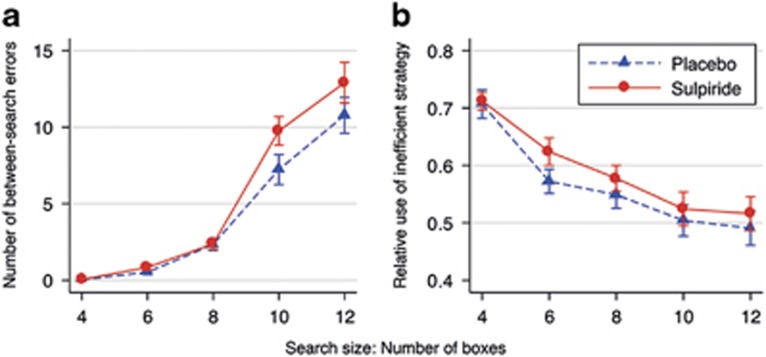
Effects of sulpiride (800 mg) on the number of between-search errors (**a**) and relative use of the inefficient strategy against task difficulty level (**b**) in the spatial working memory (SWM) task. Plotted are means±error bars of two standard errors (corrected for repeated observations).

Figure 1a shows that volunteers in the sulpiride group made more between-search errors than volunteers in the placebo group, but only in the more difficult problems. An ANOVA confirmed the significant interaction effect of drug condition with level of difficulty on between-search errors (F(4,1020)=2.66, *P*=0.031, *η*^2^=0.01). The main effect of sulpiride was nonsignificant (F(1,70)=2.15, P=0.147, *η*^2^=0.03). *Post hoc* tests confirmed that the sulpiride effects in the difficult 10-box and 12-box problems were significantly larger than the sulpiride effect in the easiest four-box problems (10-box; *P*=0.022; 12-box: *P*=0.031). The sulpiride group did not differ significantly in their strategy scores from the placebo group (F(1,70)=0.63, *P*=0.431) nor was there an interaction effect of drug condition with the task difficulty level (F(4,280)=0.40, *P*=0.807; Figure 1b). Therefore, the sulpiride effect on between-search errors cannot be explained by more frequent use of an inefficient strategy.

**Figure 2:**
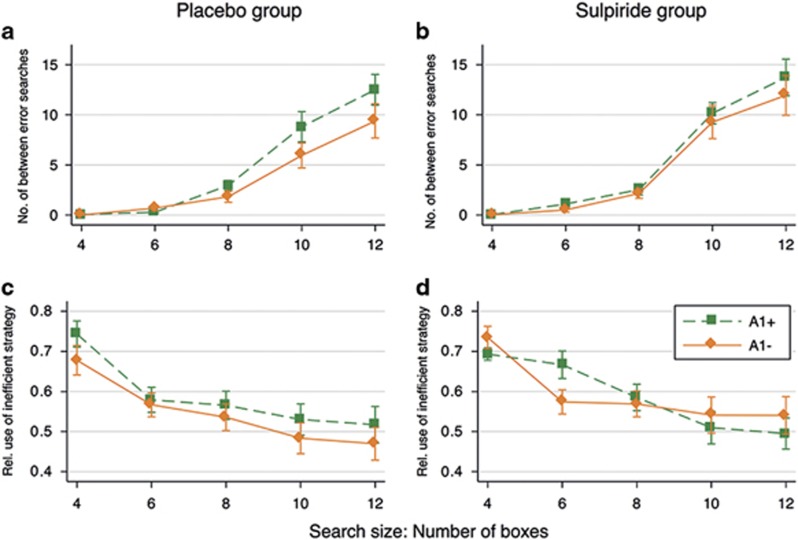
The effects of the dopamine (DA) receptor D2 Taq1A genotype on between-error searches (**a** and **b**) and relative use of the inefficient strategy against task difficulty level (**c** and **d**) in the spatial working memory (SWM) task. The left column (**a** and **c**) shows theses effects for the placebo group and the right column (**b** and **d**) for the sulpiride group only. Plotted are means±error bars of two standard errors (corrected for repeated observations).

Considering the effects of the DA receptor D2 Taq1A genotype (see Figures 2a and b), the A1− volunteers across both drug conditions made fewer between-search errors in the difficult problems than the A1+ volunteers. In the ANOVA, this was confirmed with a significant interaction effect of genotype with the level of difficulty on between-search errors (F(4,1020)=2.55, *P*=0.038, *η*^2^=0.01). *Post hoc* tests confirmed that the genotype effects in the difficult 12- and 10-box problems were significantly larger than the genotype effect in the easiest four-box problems (10-box; *P*=0.069; 12-box: *P*=0.011). The main effect of genotype was nonsignificant (F(1,70)=2.79, *P*=0.099, *η*^2^=0.04), and there was no significant interaction of genotype with drug condition (F(1,70)=0.13, *P*=0.720). For strategy scores (Figures 2c and d), the main effect of genotype was not significant (F(1,70)=0.39, *P*=0.535), nor the interactions of genotype with difficulty level (F(4,280)=0.65, *P*=0.630) or drug condition (F(1,70)=0.60, *P*=0.441). Therefore, it seems that the difference in between-search errors between the A1+ and A1− carriers cannot be explained by a difference in the extent of using an inefficient strategy. We do find, however, a significant three-way interaction of genotype with sulpiride and task difficulty level on the strategy measure (F(4,280)=2.48, *P*=0.044, *η*^2^=0.03). This effect is driven by A1+ volunteers who appear to respond differently to sulpiride in easier compared with the more difficult problems. This needs to be interpreted cautiously as we are underpowered for analysing mere genotype–behaviour associations (that is, 16–20 observations for each of the four groups).

We also tested whether sulpiride had any effect on the training process of the SWM task. We find no significant difference between the sulpiride and the placebo group in performance in the two practice trials (*P*-values >0.21). Together with the above result that there is no difference between the sulpiride and placebo group in the easy searches in the main task, this indicates that sulpiride did not affect the training process of the task.

### OTSOC measures

**Figure 3:**
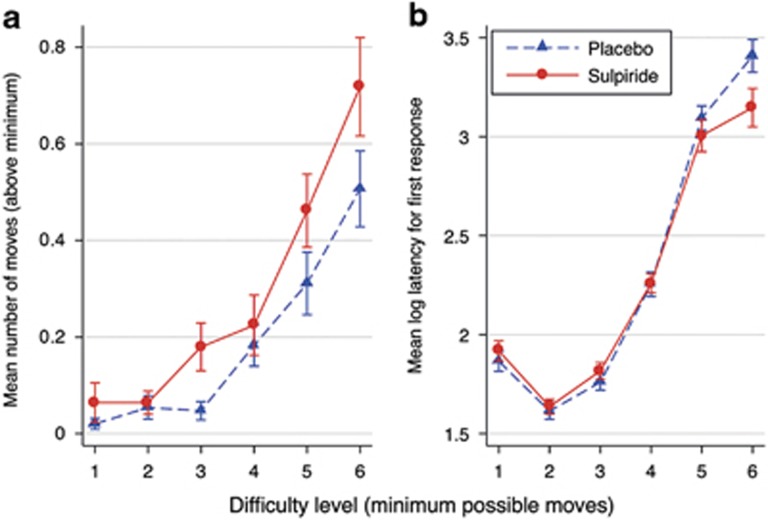
Sulpiride (800 mg) effects on the mean number of moves required to correctly solve the task (accuracy) (**a**) and the mean log response latency taken for the first response to be made against task difficulty level (**b**) in the OTSOC task. Plotted are means±error bars of 2 standard errors (corrected for repeated observations). OTSOC, one-touch stockings of Cambridge.

In the OTSOC task, volunteers in the sulpiride group required more attempts to correctly solve the task than volunteers in the placebo group (Figure 3a). In other words, the accuracy of decisions was lower in the sulpiride compared with the placebo group, which was confirmed by an ANOVA (F(1,71)=5.09, *P*=0.027, *η*^2^=0.07). The sulpiride effect on accuracy was highest for the two most difficult problems, though the interaction of task difficulty level with sulpiride was not significant (F(5,1705)=1.71, *P*=0.129). With regards to response latencies, we analysed the response latency of the first response. As can be seen from Figure 3b, there was no main effect of sulpiride on response latencies (F(1,71)=0.67, *P*=0.416). There was a significant interaction between drug condition and task difficulty on planning latency (F(5,1705)=3.43, *P*=0.004, *η*^2^=0.01), such that the response latency was significantly shorter on sulpiride compared with placebo for the most difficult problems. A *post hoc* test confirmed that the sulpiride effect on response latency was larger in the most difficult problems compared with the easiest problems (*P*=0.001).

**Figure 4:**
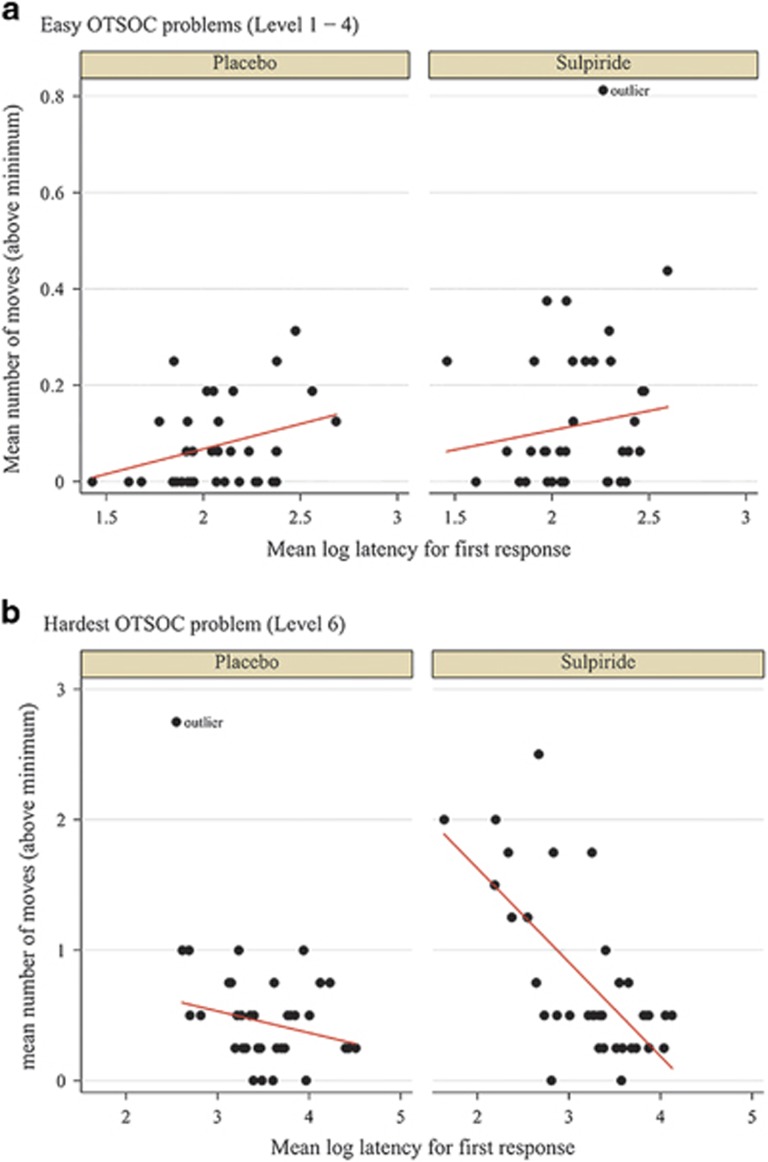
Sulpiride (800 mg) effects on the relationship between response latency and accuracy. (**a**) In this figure, the relationship is shown for the easy problems (level 1–4). (**b**) In this figure, the relationship is shown for the hardest problem (level 6). We have added a fitted line to illustrate the strength of the relationship. OTSOC, one-touch stockings of Cambridge.

To further specify the nature of this association, we tested the relationship between response latencies and accuracy for easier and more difficult problems separately. A negative relationship between speed and accuracy (represented by our measures of their opposites, response latency and mistakes) can be interpreted as a speed-accuracy trade-off. In other words, spend more time thinking about it to make fewer mistakes, or make a quick response at the cost of possibly being mistaken. To test this relationship, we use an ordinary least square regression with mean number of moves above minimum as the dependent variable and response latency, sulpiride and their interaction as explanatory variables. In the easier problems (minimum possible moves from 1 to 4), we found that the relationship between speed and accuracy was significantly positive in the placebo group (*β*_latency_=0.10, *P*=0.045) and also positive but insignificant in the sulpiride group (*β*_latency_=0.08, *P*=0.410). The interaction between sulpiride and response latency was not significant either (*β*_latency × sulpiride_=−0.02, *P*=0.834). The lack of a negative relationship between speed and accuracy indicates that there is no speed-accuracy trade-off in easy problems (Figure 4a). However, for the more difficult problems (five to six minimum possible moves), there was a negative relation between speed and accuracy in both the sulpiride (six moves: *β*_latency_=−0.72, *P*<0.001; five moves: *β*_latency_ =−0.16, *P=*0.279) and the placebo group (six moves: *β*_latency_=−0.17, *P*=0.094; five moves: *β*_latency_ =−0.27, *P*=0.089), which is consistent with a speed-accuracy trade-off (Figure 4b). The trade-off is most pronounced in the six-move problems and weaker in the five-move problems. Possibly, it takes a certain degree of complexity or difficulty for a speed-accuracy trade-off to emerge. Interestingly, in the hardest problems (six moves) the speed-accuracy trade-off was significantly larger in the sulpiride compared with the placebo group (*β*_latency × sulpiride_=−0.55, *P*<0.001). The larger speed-accuracy trade-off in the sulpiride group compared with the placebo group is driven by more fast, imprecise responses (rather than long response latencies and few mistakes) in the sulpiride group (Figure 4b). This suggests that sulpiride alters the speed-accuracy trade-off in planning towards impulsive, less accurate responses. This needs to be interpreted with caution as the number of observations in this analysis is smaller than in the main analyses reported above (that is, it is based on 74 volunteers each doing four six-move problems).

**Figure 5:**
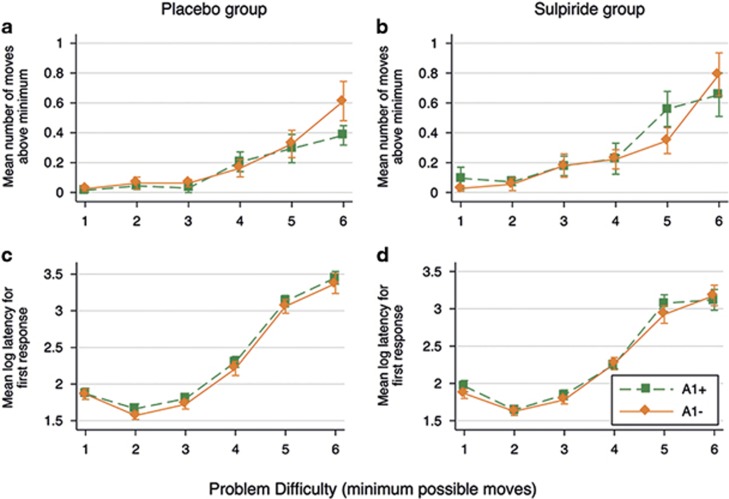
The effects of the dopamine (DA) receptor D2 Taq1A genotype on the mean number of moves required to correctly solve the task (accuracy) (**a** and **b**) and the mean log response latency taken for the first response to be made against task difficulty level (**c** and **d**) in the OTSOC task. The left column (**a** and **c**) shows theses effects for the placebo group and the right column (**b** and **d**) for the sulpiride group only. Plotted are means±error bars of two standard errors (corrected for repeated observations). OTSOC, one-touch stockings of Cambridge.

Concerning DA receptor D2 Taq1A genotype (Figure 5), there was neither a significant main effect of genotype (F(1,71)=0.01, *P*=0.941), nor a significant interaction with task difficulty (F(5,1705)=1.84, *P=*0.103) or drug condition (F(1,71)=0.85, *P*=0.361) on accuracy. Also, there was neither a significant main effect of genotype nor significant interactions of genotype with drug condition and task difficulty on response latency measures (*P*-values >0.708).

We also tested whether sulpiride had any effect on the training process of the OTSOC task. We find no significant difference in the accuracy of the decisions in the four practice trials between the sulpiride and the placebo group (*P*-values >0.19). Together with the above result that there is no difference between the sulpiride and placebo group in the one- and two-move problems in the main task, this is strong evidence that sulpiride did not affect the training process of the task.

### Prolactin secretion and side effects

Results regarding changes in prolactin levels, heart rate and blood pressure, as well as self-reported measures of sedation are outlined in the Supplementary Materials and methods as well as Supplementary Table 1.

## Discussion

We found that a high single dose of 800 mg of the selective DA receptor D2/D3 antagonist sulpiride led to significant impairments in planning accuracy on the OTSOC task, and, for the more difficult problems, on SWM in the SWM task (with no significant effect on the strategy measure). Sulpiride did not affect sensorimotor functions, as measured by response latencies in the easy problems of the OTSOC, but it speeded response latencies on the most difficult problems. We also observed significant modulatory effects of the DA receptor D2 Taq1A polymorphism on SWM, but not on planning. Sulpiride led to a significant increase in prolactin secretion, indicating postsynaptic DA receptor D2 blockade,^37, 38^ as reported previously.^24^ Further, we did not observe any significant effects of sulpiride on blood pressure, heart rate or self-reported measures of sedation.^24^

Previous studies observed that lower doses (that is, ⩽400 mg) of sulpiride-induced impairments in tasks assessing SWM using challenging tasks such as sequence generation,^7^ and the CANTAB short-term spatial recognition/location task.^23^ In contrast, lower doses of sulpiride did not induce impairments in tasks that are less challenging, such as the verbal working memory task^19^ or the easier (with a maximum of eight boxes) version of the CANTAB self-ordered SWM task.^43^ Therefore, although this categorization falls short of taking into account other differences than difficulty, it is noteworthy that we observed impairments after a high single dose of sulpiride-induced impairments specifically in the difficult (10- and 12-box), but not the easier problems of the CANTAB SWM task. Our findings therefore extend on previous results and suggest that effects may not be dose-dependent, but largely depend on task difficulty.

Performance on the SWM task can be facilitated by using a repetitive search strategy that serves to reduce the direct working memory load. This strategy utilization recruits the prefrontal cortex, as documented by imaging research as well as by a study in frontal lobe lesion patients who seem to use a relatively inefficient search strategy.^36, 44^ The fact that we found no significant effect of this high single dose of sulpiride on the strategy measure suggests that our observed impairments are unrelated to any potential impairment in prefrontal function. Rather, striatal mediation is plausible, especially given that our findings resemble those found in early stage Parkinson's disease patients, who do not show a deficit in the strategy measure either.^45^

Previous studies that used within-subject designs have reported significant interaction effects of sulpiride with administration sequence.^7, 18^ For instance, volunteers receiving sulpiride on the first day were impaired in SWM, but this was reversed in the second testing session, that is, volunteers performed better on sulpiride.^7^ Hence, although these previous findings raise interesting questions with regards to a potential role of sulpiride in learning or consolidation processes,^7^ they are difficult to interpret. Our findings that were obtained using a between-subjects design provide more conclusive evidence for a role of DA D2 receptor blockade in SWM.

With respect to planning, one study^8^ showed an improvement in planning ability and another study^7^ reported a decrease in planning ability after sulpiride administration. The latter study found this decrease in planning ability only in the most difficult problems.^7^ At the higher dose of sulpiride used here, we observed a planning impairment on both easier and more difficult problems. This divergence from earlier results could reflect a genuine dosage effect: a high single dose of sulpiride is necessary to achieve planning impairments on easier problems. However, we cannot yet definitely conclude this, given that the earlier study^7^ used a within-subject design, whereas we applied a between-subject design, the results are not strictly comparable and the differences in our results could potentially be caused by these differences in the designs applied.

Planning latencies on the OTSOC clearly increased with problem difficulty. In the most difficult problems, latencies also correlated negatively with accuracy, that is, volunteers with shorter response latencies made more mistakes, consistent with a speed-accuracy trade-off. Intriguingly, this relationship was more pronounced in the sulpiride group. In a study using a version of a task similar to the OTSOC that also requires planning of the solution in advance,^11^ the times taken to correctly solve the more difficult problems were almost twice as long compared with those observed in the original Tower of London task.^46^ This difference can be explained by the additional increase of working memory load in the OTSOC task compared with the original Tower of London task.^11^ Given that sulpiride affected working memory in our study too, the faster response latencies for difficult problems among the sulpiride group might suggest that excessive task demands caused volunteers to guess impulsively on the most difficult problems.

In our previous study on the role of the DA receptor D2 in reinforcement learning in the same cohort as the present study,^24^ a single dose of 800 mg of sulpiride had no effect on learning *per se*, but rather induced profound impairments in choice performance. These results were generally consistent with an involvement of the DA receptor D2 in tonic dopaminergic activity that has been linked to response vigour and motivational effects.^47^ Although in the present study there were no monetary rewards contingent on performance, impairments in motivation may partially explain the effects we observed, particularly those in the difficult problems. Furthermore, unlike in our earlier study where we found a modulatory role of the DA receptor D2 Taq1A polymorphism on rewarded choice performance,^24^ here we found no such differentiation of the sulpiride effect on planning ability or SWM. Such a pharmacogenetic interaction effect would have been the strongest evidence for a causal role of DA D2 receptors in these executive functions. Sulpiride, despite being one of the few relatively selective drugs affecting dopaminergic neurotransmission available for human use, has a very similar affinity for the DA D3 receptor as for the D2 receptor.^48, 49^ Therefore, our results indicate that the observed main effect of sulpiride on executive functions may also partially be mediated by DA D3 receptors. In this regard, it is interesting to note that preclinical research using D3 receptor antagonists in animals suggests a rather distinct profile compared with D2 receptor antagonists by showing positive effects on cognition.^50^ Alternatively, our findings might also echo the greater functional blockade of cortical D2/3 receptors proposed for amisulpride,^21^ compared with striatal receptor blockade.

With regards to our observed main effects of the DA receptor D2 Taq1A polymorphism, it is noteworthy that previous behavioural genetic studies have reported that the minor A1 allele (A1+) is associated with poorer performance in general cognitive ability,^51^ including visual working memory^52^ and verbal learning.^53^ A recent study^54^ that included the OTSOC as part of a larger test battery found no effect of the presence of the A1+ allele on planning. Thus, our findings are in line with those studies, showing that the presence of the A1+ allele is associated with impairments in SWM performance, but not with impairments in planning.

Although speculative, our results might therefore suggest that while a high single dose of sulpiride is required to block a sufficient number of postsynaptic DA D2 receptors to produce unambiguous deficits in planning performance, this is not the case with regards to spatial working memory performance. In the latter, both lower^7, 23^ and higher doses of sulpiride cause impairments in difficult problems suggesting that relatively low postsynaptic DA D2 receptor occupancy levels suffice to produce these impairments. The fact that these impairments are also observed in A1+ allele carriers, irrespective of sulpiride administration, further suggests that a ceiling-type of effect might exist, after which a higher number of DA D2 receptor occupancy does not lead to a more pronounced impairment in SWM. Whether DA D3 receptors represent the neuropharmacological substrate of high single dose sulpiride effects on planning performance, which would be consistent with the absence of effects of the DRD2 Taq 1a polymorphism in this and a previous study, is a subject for future pharmacogenetic studies using larger sample sizes.

In sum, we observed that a high single dose of sulpiride induces significant impairments in planning accuracy and SWM. With regards to SWM, this effect is dependent on task difficulty level. We also found that sulpiride, even when given at a high single dose, did not modulate the extent of the use of an inefficient strategy in the SWM task. However, it is unlikely that the effects of sulpiride are attributable simply to impaired sensorimotor processing, as effects in latencies varied with the level of cognitive difficulty of the tasks. Sulpiride administration speeded response latencies in the OTSOC on the most difficult problems, which might suggest that sulpiride increased impulsive guessing. Finally, we found that the presence of the A1+ allele is associated with impairments in SWM performance, but not with impairments in planning. The lack of both a main effect of the DA receptor D2 Taq1A polymorphism and an interaction with sulpiride administration on planning performance might suggest that sulpiride exerts its impact on this cognitive measure via DA D3 receptors; however, future pharmacogenetic studies using larger sample sizes need to confirm this.
