# Supplementary material for: Effects of dopamine D2/D3 receptor antagonism on human planning and spatial working memory
Source: Transl Psychiatry. 2017 Apr 25;7(4):e1107–. doi: 10.1038/tp.2017.56 (PMC5416697; doi:10.1038/tp.2017.56)
Supplement: Supplementary Information [file tp201756x1.docx]

**Supplementary Information**

# Supplementary Material and Methods

# **Plasma Prolactin Levels**

Sulpiride is well-known to induce an increase in prolactin serum concentrations by blocking DA D2 receptors which under normal conditions exert an inhibitory effect on prolactin secreting cells in the pituitary.^1^ In line with this, blood plasma prolactin levels increased significantly by 33.4 mg/ml (+ 349%) after sulpiride administration (Wilcoxon signed-ranks test, *p* = 0.000, n = 36), and this increase was significantly higher (Mann Whitney test, *p* = 0.000, n = 72) than the changes in the placebo group -0.91 mg/ml (-11%). The laboratory was not able to extract reliable prolactin data for three volunteers due to blood contamination.

# **Side-Effects**

Items in the visual analogue scales (VAS) were alert/drowsy, calm/excited, strong/feeble, muzzy/clear-headed, well coordinated/clumsy, lethargic/energetic, contented–discontented, troubled–tranquil, mentally slow/quick-witted, tense/relaxed, attentive/dreamy, incompetent/proficient, happy/sad, antagonistic/amicable, interested/bored and withdrawn/gregarious. These dimensions were presented as 10 cm lines on a computer screen and volunteers marked their current state on each line with a mouse click. In line with previous studies,^2, 3^ the factors “alertness”, “contentedness”, and “calmness” were calculated from these items.

Physiological measures as well as the VAS were measured at baseline and 3 hours after drug administration, NVL only after drug administration. Supplementary Table 1 shows all side-effects measures, their changes over time, as well as the results of a Mann-Whitney test for differences across treatment groups. NVL and VAS data of one volunteer recorded at 3 hours were lost due to a technical problem. Significance levels are not above chance level if corrected for multiple testing (Holm-Bonferroni correction). Notably, a drug-group awareness check^4^ shows that volunteers did not notice whether they got sulpiride or placebo. While 32% volunteers who received placebo believed to have received sulpiride, 35% of volunteers believed so in the sulpiride group (Mann-Whitney test, *p* = 0.81, n = 74).

Supplementary Tables

Supplementary Table 1: Side Effects of 800mg Sulpiride

| side effects | time point | N | Plac. | Sulp. | Sign. (*p*) |
| --- | --- | --- | --- | --- | --- |
| Heart rate | base | 75 | 68.6 | 67.8 | 0.966 |
|  | 3 h | 75 | 63.0 | 65.4 | 0.402 |
|  | δ | 75 | -5.6 | -2.4 | 0.538 |
| Blood pressure systolic [mm hg] | base | 75 | 131.1 | 133.2 | 0.439 |
|  | 3 h | 75 | 126.9 | 127.8 | 0.614 |
|  | δ | 75 | -4.1 | -5.4 | 0.600 |
| Blood pressure diastolic [mm hg] | base | 75 | 75.5 | 76.8 | 0.742 |
|  | 3 h | 75 | 71.5 | 70.8 | 0.762 |
|  | δ | 75 | -4.0 | -6.1 | 0.216 |
| VAS: alertness (mean) | base | 75 | 21.4 | 23.7 | 0.840 |
|  | 3 h | 74 | 24.9 | 29.0 | 0.697 |
|  | δ | 74 | 3.5 | 5.7 | 0.513 |
| VAS: contentedness (mean) | base | 75 | 22.7 | 19.8 | 0.596 |
|  | 3 h | 74 | 27.6 | 22.5 | 0.524 |
|  | δ | 74 | 4.9 | 3.2 | 0.250 |
| VAS: calmness (mean) | base | 75 | 18.3 | 24.4 | 0.874 |
|  | 3 h | 74 | 20.4 | 23.3 | 0.888 |
|  | δ | 74 | 2.1 | -0.4 | 0.996 |
| NVL: any effect | 3h | 74 | 23.0 | -38.3 | 0.709 |
| NVL: bad effects | 3h | 74 | 23.9 | -43.0 | 0.430 |
| NVL: good effects | 3h | 74 | 0.9 | -40.5 | 0.669 |
| NVL: high | 3h | 74 | -31.0 | -41.4 | 0.311 |
| NVL: rush | 3h | 74 | -42.4 | -43.5 | 0.238 |
| NVL: like drug | 3h | 74 | -38.7 | -13.8 | 0.369 |
| NVL: stimulated | 3h | 74 | -41.9 | -36.6 | 0.131 |
| NVL: performance impaired | 3h | 74 | -41.5 | -35.6 | 0.118 |
| NVL: performance improved | 3h | 74 | -16.4 | -41.2 | 0.559 |
| NVL: willing to take again | 3h | 74 | -39.3 | 4.3 | 0.593 |
| NVL: willing to pay for | 3h | 74 | -39.4 | -40.6 | 0.392 |
| NVL: active-alert-energetic | 3h | 74 | -39.3 | -37.7 | 0.940 |
| NVL: shaky/jittery | 3h | 74 | 10.5 | -36.2 | 0.236 |
| NVL: euphoric | 3h | 74 | -39.5 | -38.5 | 0.203 |
| NVL: irregular or racing heart | 3h | 74 | -33.7 | -44.6 | 0.335 |
| NVL: talkative-friendly | 3h | 74 | -43.6 | -31.5 | 0.044 |
| NVL: nauseated, queasy or sick to stomach | 3h | 74 | -42.1 | -46.5 | 0.343 |
| NVL: nervous or anxious | 3h | 74 | -44.0 | -45.0 | 0.692 |
| NVL: restless | 3h | 74 | -39.4 | -30.4 | 0.107 |
| NVL: sluggish-lazy-fatigued | 3h | 74 | -46.8 | -23.5 | 0.509 |

Notes. Base = baseline; 3h = 3 hours after drug loading; δ = difference between the value 3 hours after drug loading and the baseline; N = number of observations; Plac. = Placebo group; Sulp. = Sulpiride group; Sign. = Significance of Mann-Whitney tests for differences

Supplementary Table 2: Supplementary Table 3 Analysis of Variance of the number of errors and the strategy used in SWM

|  | *df* (n) | *df* (d) | *F* | *P* | η² |
| --- | --- | --- | --- | --- | --- |
| ***Between errors*** |  |  |  |  |  |
| Sulpiride | 1 | 70 | 2.15 | 0.147 | 0.03 |
| Genotype | 1 | 70 | 2.79 | 0.099* | 0.04 |
| Sulpiride x Genotype | 1 | 70 | 0.13 | 0.720 | 0.00 |
| Task difficulty level | 4 | 1020 | 202.26 | 0.000*** | 0.44 |
| Sulpiride x Task difficulty level | 4 | 1020 | 2.66 | 0.031** | 0.01 |
| Genotype x Task difficulty level | 4 | 1020 | 2.55 | 0.038** | 0.01 |
| Sulpiride x Genotype x Task difficulty level | 4 | 1020 | 0.49 | 0.746 | 0.00 |
| ***Rel. use of inefficient strategy*** |  |  |  |  |  |
| Sulpiride | 1 | 70 | 0.63 | 0.431 | 0.01 |
| Genotype | 1 | 70 | 0.39 | 0.535 | 0.01 |
| Sulpiride x Genotype | 1 | 70 | 0.60 | 0.441 | 0.01 |
| Task difficulty level | 4 | 280 | 50.30 | 0.000*** | 0.42 |
| Sulpiride x Task difficulty level | 4 | 280 | 0.40 | 0.807 | 0.01 |
| Genotype x Task difficulty level | 4 | 280 | 0.65 | 0.630 | 0.01 |
| Sulpiride x Genotype x Task difficulty level | 4 | 280 | 2.48 | 0.044** | 0.03 |

Notes. Repeated and Mixed-Measure ANOVA with between-search errors, and the relative strategy score as dependent variables. The number of observation is 1110 consisting of 74 volunteers taking each 15 decisions in the first estimation. The estimation with the relative strategy score as dependent variable consists of 370 observations as the strategy score is calculated for each of the 5 difficulty levels. Significance levels: <0.01 '***', <0.05 '**', <0.10 '*'

Supplementary Table 3: Analysis of Variance of the accuracy and response latency in decisions in OTSOC.

|  | *df* (n) | *df* (d) | *F* | *P* | η² |
| --- | --- | --- | --- | --- | --- |
| ***Accuracy: No. of moves above minimum*** |  |  |  |  |  |
| Sulpiride | 1 | 71 | 5.09 | 0.027** | 0.07 |
| Genotype | 1 | 71 | 0.01 | 0.941 | 0.00 |
| Sulpiride x Genotype | 1 | 71 | 0.85 | 0.361 | 0.01 |
| Task difficulty level | 5 | 1705 | 45.60 | 0.000*** | 0.12 |
| Sulpiride x Task difficulty level | 5 | 1705 | 1.71 | 0.129 | 0.00 |
| Genotype x Task difficulty level | 5 | 1705 | 1.84 | 0.103 | 0.01 |
| Sulpiride x Genotype x Task difficulty level | 5 | 1705 | 0.61 | 0.691 | 0.00 |
| ***Log latency until first response*** |  |  |  |  |  |
| Sulpiride | 1 | 71 | 0.67 | 0.416 | 0.01 |
| Genotype | 1 | 71 | 0.82 | 0.367 | 0.01 |
| Sulpiride x Genotype | 1 | 71 | 0.11 | 0.746 | 0.00 |
| Task difficulty level | 5 | 1705 | 402.61 | 0.000** | 0.54 |
| Sulpiride x Task difficulty level | 5 | 1705 | 3.43 | 0.004** | 0.01 |
| Genotype x Task difficulty level | 5 | 1705 | 0.30 | 0.912 | 0.00 |
| Sulpiride x Genotype x Task difficulty level | 5 | 1705 | 0.59 | 0.709 | 0.00 |

Notes. Repeated and Mixed-Measure ANOVA with the number of moves above the minimum number of moves or the log latency until first response as dependent variable. The number of observation is 1800 consisting of 75 volunteers taking each 24 decisions. Significance levels: <0.01 '***', <0.05 '**', <0.10 '*'

Supplementary Table 4: OLS regression of the Speed accuracy trade off in decisions in OTSOC.

| Dep. Variable Accuracy | Hard problem (level 6) | Medium problem (level 5) | Easy problems (level 1 – 4) |
| --- | --- | --- | --- |
| Log latency until first response | 0.167* | 0.266* | -0.104** |
|  | (0.0982) | (0.154) | (0.0508) |
| Sulpiride | -2.035*** | 0.184 | -0.0851 |
|  | (0.515) | (0.742) | (0.230) |
| Sulpiride * log latency | 0.554*** | -0.104 | 0.0232 |
|  | (0.142) | (0.214) | (0.110) |
| Constant | 4.967*** | 4.835*** | 6.140*** |
|  | (0.352) | (0.537) | (0.103) |
| Observations | 74 | 75 | 74 |
| R^2^ | 0.458 | 0.080 | 0.080 |

Notes. Ordinary least square (OLS) regression with accuracy as dependent variables. Accuracy is defined as the average number of moves above the minimum number of moves in the various difficulty levels. The table reports the regression coefficients with robust standard errors in parenthesis. Significance levels: <0.01 '***', <0.05 '**', <0.10 '*'.

Supplementary References

1. Muller EE, Locatelli V, Cella S, Penalva A, Novelli A, Cocchi D. Prolactin-Lowering and Prolactin-Releasing Drugs Mechanisms of Action and Therapeutic Applications. *Drugs* 1983; **25**(4)**:** 399-432.

2. Chamberlain SR, Müller U, Blackwell AD, Clark L, Robbins TW, Sahakian BJ. Neurochemical modulation of response inhibition and probabilistic learning in humans. *Science* 2006; **311**(5762)**:** 861-863.

3. Eisenegger C, Knoch D, Ebstein RP, Gianotti LRR, Sandor PS, Fehr E. Dopamine receptor D4 polymorphism predicts the effect of L-DOPA on gambling behavior. *Biol Psychiatry* 2010; **67**(8)**:** 702-706.

4. Eisenegger C, Naef M, Snozzi R, Heinrichs M, Fehr E. Prejudice and truth about the effect of testosterone on human bargaining behaviour. *Nature* 2010; **463**(7279)**:** 356-359.
